# Impact of sin taxes on consumption volumes of sweetened beverages and soft drinks in Saudi Arabia

**DOI:** 10.12688/f1000research.25853.2

**Published:** 2021-01-14

**Authors:** Rania Megally, Ayoub Al-Jawaldeh

**Affiliations:** 1 Economics, British University in Egypt, Giza, 11837, Egypt; 2 Regional Office for the Eastern Mediterranean (EMRO), World Health Organization (WHO), Cairo, 11371, Egypt

**Keywords:** Saudi Arabia, Health and Economic Development, Behavioral Economics, Health Behavior, Welfare Economics, Household Behavior and Family Economics, Development Planning and Policy, Economy wide Country Studies

## Abstract

**Background: **The consumption of sweetened drinks plays a major role in increasing the burden of diseases such as obesity and cardiovascular diseases. The prevalence rate of obesity and overweight individuals in Saudi Arabia has increased alarmingly among children over the past decade, revealing a crucial need the initiate and monitor effective prevention measures of obesity. Hence, this paper aims to measure the impact of sin taxes of sugar-sweetened beverages on the consumption level of such beverages in Saudi Arabia. Building enough evidence to guide other countries in the Eastern Mediterranean Region (EMR) on how to reduce the level of sugar intake consumption to reduce obesity and malnutrition has an impact on the economy as a whole.

**Methods: **The excise taxes on sugar-sweetened beverages were applied in 2017. The impact of this was measured using a time series data set that covered sales volumes of soft drinks in Saudi Arabia from 2010 to 2020.

**Results: **Sin taxes had a significant negative impact on the sales volume over the years. A significance level of <1% was found as sales volume of soft drinks decreased by 57.64% from 2010 to 2017 when sin taxes were applied on energy and soft drinks.

**Conclusions: **Sin tax policy is recommended to other countries in the EMR to reduce obesity levels among children. These recommendations advocate the recommended priority actions by the World Health Organization for the strategy on nutrition for the EMR 2020-2030.

## Introduction

Sugar-sweetened beverages are well-known to be related to adverse health outcomes as they are considered a significant source of excess caloric intake ( Fidler Mis *et al.*, 2017). The consumption of such sweetened drinks, especially those containing fructose, plays a major role not only in increasing the calories, but also in increasing the burden of diseases such as obesity, cardiovascular diseases, fatty liver disease, and increased blood pressure. Multiple problems can occur in obese children, such as the high risk of having sleep disordered breathing ( Paglia *et al.*, 2019). Further, consuming large quantities of sugar is associated with adverse health outcomes such as attention deficit disorder and hyperactivity disorder ( Del-Ponte *et al.*, 2019; Johnson *et al.*, 2011). Children are categorized as overactive when they cannot sit still, cannot concentrate, cannot keep silent, and leave one unfinished activity and move to the next ( Bellisle, 2004).

The impact of sugar-sweetened beverages has been also measured on children’s cognitive level using the Kaufman Brief Intelligence Test. The results showed that as the consumption of sugar-sweetened beverages increases, the verbal scores of the mid-childhood Kaufman test decreases. On the contrary, there was a positive association between fruit consumption and higher cognitive scores ( Cohen *et al.*, 2018). These facts demonstrate the negative impact of sugar-sweetened beverage consumption on the cognition level of the children, showing that it is directly related to their productivity in schooling performance, which is eventually reflected in their productivity and income level in the future.

The increased risk of gaining weight caused by the sugar contained in many drinks, such as soft drinks and energy-rich drinks, has been proved in many longitudinal studies and clinical trials. It should be noted that there were no significant differences in gaining weight recorded when sucrose was replaced with low-calorie artificial sweeteners. However, some of the artificially sweetened drinks can increase the risk of diabetes and may lead to weight gain and obesity ( Al-Jawaldeh *et al.*, 2018).

Eastern Mediterranean Region (EMR) policymakers have been advised to measure the levels of current free-sugar intake in drinks and foods given the emphasized importance of reducing sugar intake in areas where there is malnutrition. Also, the adoption of measures and policies became a necessity given that 60% of our daily energy intake is constituted from carbohydrates, such as refined cereals and sugars ( Al-Jawaldeh *et al.*, 2018). 

The high prevalence of obesity and overweight adults and children has been observed in the EMR, with the highest prevalence of regional diabetes rates worldwide. These facts spotlight the high rates of overweight children in the region as it has been observed in some countries that more than 15% of children are affected ( World Health Statistics, 2016).

Saudi Arabia is among the Gulf Cooperation Council (GCC) countries that has experienced a high prevalence rate of obesity and overweight; reaching 13.4% and 18.2% of overweight and obesity, respectively ( Alqarni, 2016). The prevalence rate of obesity and overweight individuals has increased alarmingly among children in Saudi Arabia over the past decade, revealing a crucial need to initiate and monitor effective prevention measures of obesity ( Al-Hussaini *et al.*, 2019). 

Mexico reached the highest consumption level of sugar-sweetened beverages worldwide in 2012 ( Valadez, 2013), which was linked to the high prevalence rates of obesity and overweight individuals – 30% of children and 71% of adults ( Barquera *et al.*, 2013; Encuesta Nacional de Salud y Nutrición (2012). Evidence showed that 71% of the consumption of added sugars was derived from sugar-sweetened beverages ( Sánchez-Pimienta *et al.*, 2015). The Mexican government reacted by setting sugar-sweetened beverages taxes in order to reduce their consumption, leading to a reduction in the high rate of obesity/overweightness. In addition, studies showed that such taxes decreased the consumption of sugar-sweetened beverages in Mexico ( Colchero *et al.*, 2016; Pan American Health Organization, 2015). 

## World Health Organization recommendations and fiscal policies

Resilient and sustainable food systems for healthy diets are one of the main nutrition strategies of the United Nations Action on Nutrition. The EMR of the World Health Organization (WHO) has developed an action plan and policy statement for sugar reduction based on the guidelines of the WHO, considering the energy intake per person that exceeded 2000 kcals/day in all regional countries ( Alwan *et al.*, 2017). Hence, the average intake of sugar should be decreased by more than 50% for adults and children ( WHO, 2020).

The WHO recommends the use of well-designed subsidies and taxes in order to incentivize the consumption and production of healthier drinks and foods. One of the vital Eastern Mediterranean regional initiatives that have been developed to support the actions for obesity prevention 2019–2023 is the implementation of fiscal measures. These fiscal measures include the implementation of applying taxes on sugar-sweetened drinks, as well as other subsidies and taxes that promote healthier diets ( WHO, 2019). 

### Objective

The objectives of this study were as follows:

1. Provide an overview of the impact of interventions to discourage sugar intake and reduce the consumption level;
2. Measure the impact of sin taxes on the sales and consumption level of sugar sweetened beverages in Saudi Arabia, one of the countries in the Mediterranean region who applied excise taxes on these products.

## Methods

### Data collection

This paper measured the impact of sin taxes on sugar-sweetened beverages using a time series data set that covered sales volumes of soft drinks in Saudi Arabia from 2010 to 2020. The data were secondary data collected by Global Company Intelligence (GCI), which is a company that specializes in collecting data from national governments and international industrial companies.

The authors requested GCI to create a report with the following variables concerning Saudi Arabia for the period 2010 to 2020: value of soft drinks in million dollars and local currency of Saudi Arabia per year, consumption of soft drinks in million liters per year, percentage growth from previous period to current period in million liters, and percentage growth from previous period to current (PP Growth %). The dataset created by GCI can be found in *Underlying data*. The impact of sin taxes has been tested via the following model:

$$SalesVol_{t}=\beta_{0}+\beta_{1}\;Price_{t}+\varepsilon$$

Where SalesVol _t_ refers to the sales volume in million liters and Price _t_ refers to the price of soft drinks in million US dollars.

### Data analysis

STATA 16.0 was used to conduct descriptive statistics and data anlaysis. Subsequently autocorrelation of the sales volume trends over the years was tested. Finally, the impact of sin taxes on sales volumes was been tested via regression analysis after testing for normal distribution of the time series of both dependent and independent variables using the Shapiro-Wilk test.

## Results

In Saudi Arabia, the obesity rate has doubled over the past decade ( Al-Hussaini *et al.*, 2019). This lead to vital actions taken by Saudi policymakers, such as imposing 50% excise taxes on sweetened-soft beverages in 2017, as a response to the proposed policy priorities that have been recommended by the EMR- WHO to prevent diabetes and obesity in the region ( Alwan *et al.*, 2017).

Table 1 shows the decline in percentage change of sugar-sweetened drink consumption and percentage change in sweetened juice consumption due to imposed excise taxes, from 2016 to 2019.

**Table 1.** Trends of percentage growth from previous period (PP growth) of sugar-sweetened beverages (SSB) and sweetened juice in Saudi Arabia.

Year	Excise tax on SSB	PP growth of SSB (%)	PP growth of sweetened juice (%)
**2016**	0%	5.44	-2.64
**2017**	50%	1.33	-12.85
**2018**	50%	2.52	-8.52
**2019**	50%	2.34	-8.29

### Estimating the impact of sin taxes on the change in sales volume over years

**Figure 1.**
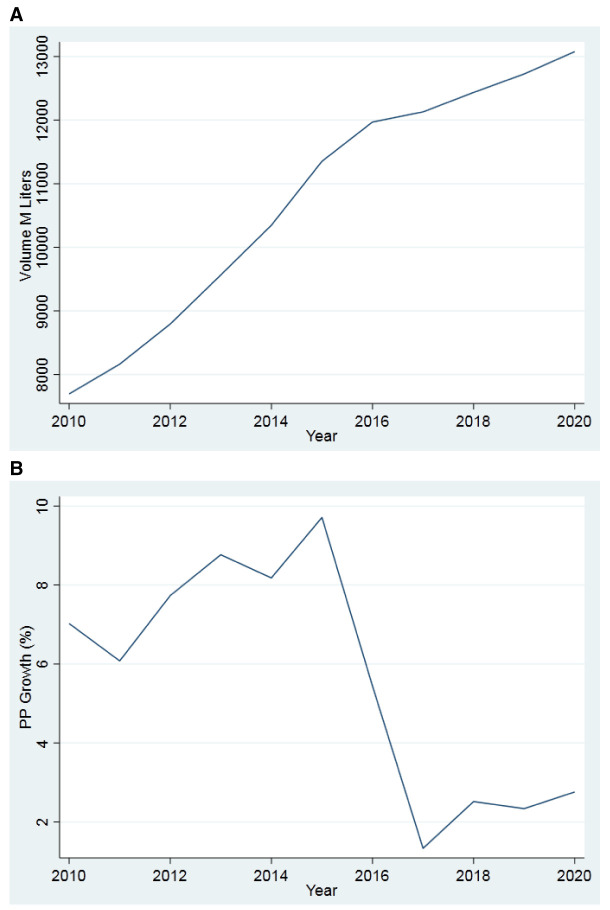
Sales volume of soft drinks in Saudi Arabi from 2010–2020. ( **a**) Sales volume in million liters; ( **b**) percentage growth from previous period (PP growth %).

From 2010 to 2017, sales volumes of soft drinks decreased by 57.64%; there was a decrease from 7694.6201 to 12129.507 million liters annually during the period when sin taxes have been applied to energy and soft drinks. Saudi Arabia started advocacy and communication campaigns before introducing sin taxes as part of their national action plan for obesity prevention, guided by the National Food Based Dietary Guidelines, which had key messages to social media, TV advertisements, and direct communication through schools ( WHO, 2019). This led to slight reduction up to 2016; however, there was a sharp significant reduction after imposing sin taxes in 2017. Overall, an increasing trend in sales volume of soft drinks over the last decade has occurred ( Figure 1a); however, the percentage change of the sales volume (PP growth) started to decrease sharply in 2017, the year that sin taxes were applied to the prices of soft drinks ( Figure 1b). 

### Testing autocorrelation over time

In Table 2, autocorrelation shows that the time series has been divided into three lags indicating three stages of PP growth. The results show that the impact of sin taxes in reducing sales volume over time and the trend between the three lags is statistically significant (P<0.05).

**Table 2.** Time lags of sales growth of soft drinks in Saudi Arabia 2010–2020, as analysed by autocorrelation.

					-1 0 1	-1 0 1
Lag	AC	PAC	Q	Prob>Q	Autocorrelation	Partial Autocor
**1**	**0.6988**	**0.7766**	**6.5117**	**0.0107**	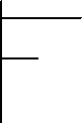	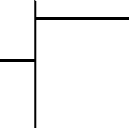
**2**	**0.3010**	**-0.3193**	**7.8707**	**0.0195**		
**3**	**-0.0157**	**-0.0357**	**7.8749**	**0.0487**		

### Testing normal distribution of the variables

The normal distribution of sales volume, the growth rate of sales volume, as well as the value of soft drinks have been tested before estimating a regression model using the Shapiro Wilk test. H _0_ assumes a normal distribution of the variables. Table 3 shows that the time series of all variables are normally distributed, which qualifies them to be used in the regression models.

**Table 3.** Shapiro Wilk testing normal distribution of variables.

Variable	Observations	Prob > z
Volume (in million liters)	11	(> 0.10)
PP Growth	11	(> 0.10)
Value (in million dollars)	11	(> 0.10)

The results show that sin taxes have a significant negative impact on the percentage volume growth over the years with a significance level <1% and high R ^2^ of around 68%, which reflects that the model estimates the impact of sin taxes at 68% ( Table 4).

**Table 4.** Estimating the impact of sin taxes on sales volume of soft drinks in Saudi Arabia using regression model.

	PP Growth	95% Confidence Interval	P-Value
Value (in million dollars)	-0.0011593	(-0.0017579, -0.0005607)	<0.01
Constant	14.2385	(9.633518, 18.84348)	<0.01

## Discussions

The prevalence rate of obesity and overweightness has increased alarmingly among children in Saudi Arabia over the past decade, leading to a crucial need for intervention ( Al-Hussaini *et al.*, 2019). Evidence shows that there is a positive relationship between the consumption of sugar-sweetened beverages and the prevalence rate of obesity and overweightness ( Paglia *et al.*, 2019). Hence, EMR of the WHO recommended governments to introduce a sustainable reduction of sugar intake over the coming 3–4 years. Considerable reductions of sugar intake should be 50% or more to terminate the increase in obesity and diabetes to decrease the burden of premature deaths, as non-communicable diseases are expected to reach 25% by 2025 ( WHO, 2020). These facts were alarming enough for the WHO to set their action plans for the nutrition strategy 2020–2030 ( WHO, 2019), which recommended the implementation of taxes on sugar-sweetened beverages to reduce obesity rates in various nations.

These facts were inspiring enough to study the impact of sin taxes that have been applied to sugar-sweetened beverages in one EMR country, Saudi Arabia, in 2017. The current study is consistent with the regional WHO recommendations to apply the United Nations Political Declaration on Non-Communicable Diseases, the priority legal interventions that target the prevention of non-communicable diseases in the EMR ( Gostin *et al.*, 2017), as well as the recommendations of the Commission on Ending Obesity ( Food and Agriculture Organization of the United Nations/World Health Organization, 2017; Report of the Commission on Ending Childhood Obesity, 2016).

The results showed that the rate of change in the sales volume over the last decade in Saudi Arabia started to decrease sharply in 2017, the year the sin taxes have been applied to the prices of soft drinks. Sales volumes from 2017 were increasing but at decreasing rates, and the sin tax had a significant negative impact on the change of sales volumes over the past 10 years. These results are in line with the nutrition strategies of the United Nations of Action on Nutrition that was based on evidence and experimental studies, which observed effective fiscal measures of taxes and subsidies in shifting habits of purchases and promotion of dietary change ( Thow *et al.*, 2014; WHO, 2015a; WHO, 2016). This is expected to decrease the obesity levels among Saudi children over the coming years. Improvements in children’s health are expected to be reflected in better cognition, intelligence, and schooling performance of the children. Moreover, this study showed a decrease of around 57% of the percentage change of sales volume, which aligns with previous evidence from countries that applied taxes and reduced the purchases of sugar-sweetened beverages in a range of 20–50% ( Colchero *et al.*, 2016; Ells *et al.*, 2015; Mozaffarian *et al.*, 2012; Powell *et al.*, 2013; Thow *et al.*, 2014; WHO, 2015b; WHO, 2016).

Limitations of this research were not knowing the nutrition status of the children after imposing the sin taxes, and limited analysis using simple linear regression that does not consider any confounding factors.

## Conclusions and recommendations

Recently, the WHO shed light on the importance of reducing obesity among children in the EMR given the high rate that has been observed lately. One intervention that was suggested is to impose taxes to sugar-sweetened beverages ( Lobstein, 2014). The impact of applying such an intervention has been previously shown to have a positive impact in reducing the level of consumption of sugar-sweetened beverages in GCC countries. Hence, such a policy is recommended to extend to cover other countries in the EMR. In addition, public health education should be considered using social marketing campaigns and restriction of media advertisements about sugar-sweetened beverages, and guide the manufacturers to impose compulsory information of health risks on the front of pack labels of SSBs. This should support the imposition of sin taxes, as recommended by Lobstein (2014). In addition, schools and pre-schools should support such policy by encouraging the consumption of water and discouraging the intake of sweetened beverages that should not be available by educational and behaviour changing programs. Such actions should be supported by parents who should abide the availability of SSBs at home and avoid its consumption to please their children. These recommendations advocate the recommended priority actions by the WHO for the strategy on nutrition for the EMR 2020-2030 ( WHO, 2019).

## Data availability

### Underlying data

Harvard Dataverse: Soft Drinks Volumes, https://doi.org/10.7910/DVN/9SE4V3 ( Megally, 2020).

Data are available under the terms of the Creative Commons Zero "No rights reserved" data waiver (CC0 1.0 Public domain dedication).
