# Supplementary material for: Impact of sin taxes on consumption volumes of sweetened beverages and soft drinks in Saudi Arabia
Source: F1000Res. 2021 Jan 14;9:1117. Originally published 2020 Sep 10. [Version 2] doi: 10.12688/f1000research.25853.2 (PMC7809884; doi:10.12688/f1000research.25853.2)
Supplement: Peer review history

# Peer review: Reviewer response for version 1

**DOI:** 10.5256/f1000research.28531.r71181

**Published:** 2020-10-14

**Authors:** Ali Arabi

**Affiliations:** 1 Faculty of Veterinary Medicine, University of Khartoum, Khartoum, Sudan

The introduction:

- There are many repeated phrases concerning obesity and overweight - the authors need to shorten.
- An introduction should include other problems like caries and enamel erosion, short sleep duration, hyperactivity, increased blood pressure and non-alcoholic fatty liver disease. High sugar content and low pH render SSB is a real threat to the developing dentition.

Methodology and results are okay.

 In recommendations the authors should also include:

- Advertising of SSBs to children should be banned. 
- The introduction of compulsory information on the front of pack labels of SSB on health risks should be considered.  

*At School:*

- Educational and behaviour-changing programs on appropriate choices of beverages and encouraging water consumption should be implemented in pre-schools and schools.
- Pre-schools and schools should offer unlimited access to drinking water, for example, by water fountains, whereas SSBs and other sweet beverages should not be made available. 

*Parents:*

- Parents should avoid the use of SSBs to please their children. 
- SSBs should not be easily available at home. 

Is the work clearly and accurately presented and does it cite the current literature?

Yes

If applicable, is the statistical analysis and its interpretation appropriate?

I cannot comment. A qualified statistician is required.

Are all the source data underlying the results available to ensure full reproducibility?

Yes

Is the study design appropriate and is the work technically sound?

Yes

Are the conclusions drawn adequately supported by the results?

Yes

Are sufficient details of methods and analysis provided to allow replication by others?

Yes

Reviewer Expertise:

gastroenterology and nutrition

I confirm that I have read this submission and believe that I have an appropriate level of expertise to confirm that it is of an acceptable scientific standard.

# Response

**Published:** 2020-12-01

**Authors:** Rania Megally

**Affiliations:** Independent Consultant, Egypt

Dear Dr. Ali, 

 First of all, I do like to express my gratitude for your thorough review of our paper that was of a great benefit as we have considered all your comments in the updated version of the paper

 Thank you again for your sincere efforts 

 Best regards, 

 Rania

# Peer review: Reviewer response for version 1

**DOI:** 10.5256/f1000research.28531.r71182

**Published:** 2020-09-21

**Authors:** Maha Hoteit

**Affiliations:** 1 Faculty of Public Health, Nutrition and Dietetics Department, Lebanese University, Hadath, Lebanon

Sin tax is a tool that contribute to enhancing consumer behavior and if implemented can have a positive health impact on the population. In general, the decisions taken at the governmental level in terms of the introduction of new taxes, should be supported by reliable analyses and this paper is a good evidence to show the improvement of the countries to reduce the intake of sugar through the decrease in consumption of soft drinks that is responsible of aggravating the prevalence of obesity in EMR. The results of this study highlighted the sharp decrease  in 2017, the year when the sin taxes have been applied to the prices of soft drinks and the sin tax had a significant negative impact on the change of sales volumes over the past 10 years. This manuscript is well written that reflect background literature on soft drink consumption in Saudi Arabia and highlight the importance of implementing sin taxes in all GCC.

Is the work clearly and accurately presented and does it cite the current literature?

Yes

If applicable, is the statistical analysis and its interpretation appropriate?

Yes

Are all the source data underlying the results available to ensure full reproducibility?

Yes

Is the study design appropriate and is the work technically sound?

Yes

Are the conclusions drawn adequately supported by the results?

Yes

Are sufficient details of methods and analysis provided to allow replication by others?

Yes

Reviewer Expertise:

Public Health Nutrition, Food composition tables, food microbiology, autism and nutrition, community nutrition

I confirm that I have read this submission and believe that I have an appropriate level of expertise to confirm that it is of an acceptable scientific standard.

# Response

**Published:** 2020-11-25

**Authors:** Rania Megally

**Affiliations:** Independent Consultant, Egypt

Dear Dr. Maha, 

 I just do like to thank you for your sincere review and comments that we the authors do highly appreciate 

 Many thanks, 

 Best regards, 

 Authors
